# Bone Tissue Engineering and Nanotechnology: A Promising Combination for Bone Regeneration

**DOI:** 10.3390/biology13040237

**Published:** 2024-04-02

**Authors:** Luana Vittoria Bauso, Valeria La Fauci, Clelia Longo, Giovanna Calabrese

**Affiliations:** Department of Chemical, Biological, Pharmaceutical and Environmental Sciences, University of Messina, Viale Ferdinando Stagno d’Alcontres, 31, 98168 Messina, Italy; valeria_lafauci@outlook.it (V.L.F.); clelia.longo@studenti.unime.it (C.L.)

**Keywords:** large bone defects, bone tissue engineering, biomaterials, nanotechnology, nanoparticles, bone regeneration

## Abstract

**Simple Summary:**

Bone tissue engineering is one of the most promising approaches for the restoration of large bone defects. Nevertheless, to date, several disadvantages limit its use due to an inability to completely fulfill all the clinical needs. In this context, in recent years, the application of nanotechnology to improve the mechanical, chemical–physical, and biological properties of biomaterials for bone tissue engineering has received great interest from researchers. Nanomaterials, including nanoparticles, are the key elements of such nanotechnologies due to their high penetrating ability and surface area, mechanical strength enhancement, improved cell adhesion, differentiation, and growth, enhanced antibacterial properties, and biocompatibility. In this review, we report on the latest in vitro and in vivo studies on the combination of nanotechnology and bone tissue engineering as promising approach for the regeneration of large bone defects.

**Abstract:**

Large bone defects are the leading contributor to disability worldwide, affecting approximately 1.71 billion people. Conventional bone graft treatments show several disadvantages that negatively impact their therapeutic outcomes and limit their clinical practice. Therefore, much effort has been made to devise new and more effective approaches. In this context, bone tissue engineering (BTE), involving the use of biomaterials which are able to mimic the natural architecture of bone, has emerged as a key strategy for the regeneration of large defects. However, although different types of biomaterials for bone regeneration have been developed and investigated, to date, none of them has been able to completely fulfill the requirements of an ideal implantable material. In this context, in recent years, the field of nanotechnology and the application of nanomaterials to regenerative medicine have gained significant attention from researchers. Nanotechnology has revolutionized the BTE field due to the possibility of generating nanoengineered particles that are able to overcome the current limitations in regenerative strategies, including reduced cell proliferation and differentiation, the inadequate mechanical strength of biomaterials, and poor production of extrinsic factors which are necessary for efficient osteogenesis. In this review, we report on the latest in vitro and in vivo studies on the impact of nanotechnology in the field of BTE, focusing on the effects of nanoparticles on the properties of cells and the use of biomaterials for bone regeneration.

## 1. Introduction

Bone remodeling is a physiological process which requires a dynamic balance between the osteoblastic activity that produces new bone and bone resorption mediated by osteoclasts [1]. Since bone is a self-healing tissue, small skeletal defects are generally repaired on their own, while in large bone lesions, bone regeneration is impaired [2]. Severe bone defects due to trauma, aging, osteoporosis, degenerative disorders (osteoarthritis), autoimmune conditions (rheumatoid arthritis), or tumor removal [3] are a major cause of disability worldwide [4], affecting approximately 1.71 billion people [5]. Therefore, it is widely recognized that this condition constitutes a true public health emergency, given the considerable financial and social costs resulting from it, particularly regarding the management of pathological fragility fractures, which are more often misdiagnosed, leading to severe morbidity or even death [6]. Currently, autografts, allografts, and xenografts represent the traditional surgical approaches for large bone lesions, although many disadvantages limit their use [7,8]. As a result, it is crucial to investigate novel potential therapeutic strategies that may improve the quality of life of patients, avoiding side effects, including pain, donor site morbidity, rejection, transmission of diseases, and high cost [9]. In this context, the tissue engineering (TE) approach could represent the most promising alternative for bone repair by overcoming these limitations and addressing clinical needs [10]. Bone tissue engineering (BTE) is an innovative and promising alternative to treat bone defects based on the development of biomaterials that support tissue regeneration. Although in recent years, numerous efforts have been made to generate biomaterials that are capable of satisfying all clinical needs, unfortunately, to date, no ideal material has been discovered. For this reason, great attention has been paid to nanotechnologies, thanks to the possibility of generating nanoengineered particles to improve the properties of the scaffold, such as mechanical strength and controlled release of growth factors [11,12]. NPs obtained from various types of materials such as ceramics, metals, and natural and synthetic polymers have been widely investigated as possible candidates for BTE due to their high penetrating ability and surface area, mechanical strength enhancement, improved cell adhesion, differentiation, and growth, enhanced antibacterial properties, and biocompatibility [13].

This review discusses recent advances in the field of BTE, focusing on the promising role of nanotechnology and on the interaction of NPs with osteoprogenitor cells and biomaterials, in terms of improving the chemical–physical, mechanical, and biological properties. Therefore, the aim of this overview is to highlight how the combination of nanotechnology and BTE can open new doors in the field of bone regeneration, thus offering an innovative solution for the treatment of large bone defects.

## 2. Conventional and Innovative Bone Graft Approaches

Autografts consist of tissue transplantation from one site to another within the same person. It represents the “Gold Standard” for treating severe bone defects, offering all three of the requirements for bone regeneration: osteoconductivity, osteoinductivity, and osteogenicity [14]. Nonetheless, there are significant disadvantages to the use of autografts, including high costs, donor site morbidity, bleeding, inflammation, infection risk, and persistent post-operative pain [15]. Allografts are the second most common bone-grafting approach, involving transplanting bone tissue from human donors, often cadavers. Compared to autografts, allografts are correlated with risks of immune rejection, infection, and disease transmission [16]. Additionally, they have poor osteoinductive properties and no cellular components, because donor bone is devitalized to avoid immunological rejection [17]. Xenografts involve the transplantation of bone tissue from nonhuman species, especially from bovines [18]. Therefore, compared to allografts, xenografts are much more antigenic and necessitate more sterile processing, which may result in reduced osteoinductive properties. On the other hand, this kind of graft may be more economical and easily accessible due to the large availability of donors [19]. Synthetic grafts include artificial materials, also named bone substitutes, that can be classified as first and second generation implants [20]. First generation of implants, developed in the 1960s, use bioinert and non-biodegradable materials that integrate with host tissue without triggering an immune response or promoting bone regeneration. These included metals (e.g., stainless steel, titanium, or cobalt-chromium alloys), ceramics such as aluminium oxide (alumina), zirconium oxide (zirconia), and carbon, synthetic polymers like silicone, poly-ethylene (PE), polyurethanes (PU), polypropylene (PP), polymethylmethacrylate (PMMA), and acrylic resins [21]. Second generation implants were developed between 1980 and 2000 to improve both biocompatibility and biodegradation. They comprise naturally derived (e.g., collagen, hyaluronic acid) and synthetic polymers, such as polycaprolactone (PCL), polylactide (PLA), and polyglycolide (PGA), calcium phosphates (CaP), including hydroxyapatite (HA) and beta-tricalcium phosphate (b-TCP), calcium carbonate, calcium sulfates (CaS), and bioactive glasses (silica or non-silica based) [22]. Although first and second generation implants have a reduced risk of disease transmission and immune rejection for the patient, they are not considered ideal materials, as they lack osteoinductive and osteogenic properties and are highly susceptible to bacterial infections [23,24]. Therefore, considering the non-negligible limitations associated with conventional bone grafting procedures, there is currently no effective surgical strategy for the repair of large bone defects. Consequently, this clinical need has stimulated the development of alternative and innovative strategies based on TE approaches for bone regeneration. The term “tissue engineering” was introduced in 1988 at a National Science Foundation workshop to indicate “an interdisciplinary field which applies the principles of engineering and life sciences towards the development of biological substitutes that aim to maintain, restore or improve tissue function” [25]. TE is a branch of regenerative medicine which requires the use of three-dimensional biomaterials, also known as scaffolds. Specifically, BTE implicates the development of a scaffold which is able to mechanically support cell recruitment, adhesion, proliferation, differentiation, and extracellular matrix (ECM) formation for bone tissue regeneration [26]. Scaffold bioactivity depends on its mechanical, structural, and chemical–physical properties, as well as its intrinsic osteoconductivity [27].

In BTE, three key factors are required for successful bone regeneration: (1) osteoprogenitor cells, including embryonic stem cells (ESCs) and mesenchymal stem cells (MSCs) which are able to form a functional matrix [28]; (2) specific growth factors that stimulate cell migration, proliferation, differentiation, and vascularization [29], such as bone morphogenetic proteins (BMPs) [30], trans-forming growth factor β (TGF-β) [31], insulin-like growth factors I and II (IGF-I/IGF-II) [32], vascular epithelial growth factor (VEGF) [33], fibroblast growth factor (FGF) [34], and platelet-derived growth factor (PDGF) [35]; and (3) biomaterials that offer a three-dimensional (3D) matrix for cell adhesion and growth [36,37].

**Figure 1:**
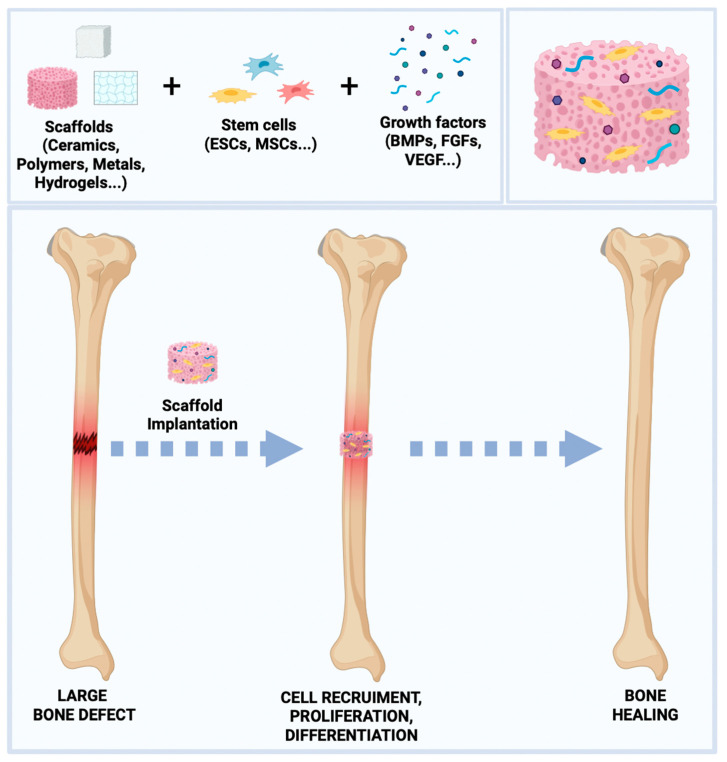
Key factors of BTE and the bone regeneration process.

Figure 1 shows the BTE approach.

## 3. Biomaterials for BTE Applications

The term “biomaterial” was first defined as “any substance, other than a drug, or a combination of substances, synthetic or natural in origin, which can be used for any period of time, as a whole or as a part of a system, which treats, augments or replaces any tissue, organ or function of the body” at the Consensus Development Conference on the Clinical Application of Biomaterial (Bethesda, MD, USA) in 1982 [38].

In this regard, one of the main goals of BTE is to develop biomaterials with appropriate biological features, i.e., biocompatibility (the ability to promote cell adhesion, proliferation, and migration) [39], biodegradability (the ability to be degraded into non-toxic byproducts that are easily eliminated by the body without interfering with other tissues) [40], non-immunogenicity (the ability to not trigger rejection by the host’s immune system) [41], anti-bacterial properties (the ability to reduce biofilm formation, thereby avoiding infection risk and antimicrobial resistance) [42], osteogenesis (the formation of new bone) [43], osteoconduction (providing structural support to promote host tissue recovery) [44], and osteoinduction (the recruitment of stem cells from the biological environment to induce osteogenic differentiation) [45].

In order to achieve the aforementioned features, biomaterials must possess adequate structural properties in terms of the scaffold 3D architecture and porosity that influence cell adhesion and survival, as well as suitable mechanical properties, such as strength and stiffness, which affect cell proliferation and differentiation [46,47].

The most widely used biomaterials for the development of BTE scaffolds are classified as follows:

(a) Ceramics are the most promising biomaterials due to their good mechanical properties and excellent biocompatibility [48]. They include bioglass, alumina, zirconia, and CaP-based materials such as HA, b-TCP, and biphasic calcium phosphate (BCP) [49,50]. They can accurately imitate the ECM composition of natural bone [51], thereby improving osteoblastic proliferation and differentiation [52], and their biodegradability allows the release of ions that can contribute to bone tissue regeneration [53]. Furthermore, ceramic biomaterials provide highly interconnected porous structures that enable neo-vascularization, cell migration, and bone growth [54]. In their study, Mondal et al. demonstrated that fish scale-derived natural HA (FS-HAp) scaffolds successfully mimicked the cancellous/cortical bone system in terms of structure, porosity, and mechanical strength and exhibited excellent bioactive behavior. Furthermore, in vitro and in vivo studies by those authors suggested that these scaffolds could provide osteoconductive support, facilitating new cell growth on their surface [55]. In their in vitro and in vivo study, Jiao et al., investigated the osteogenic and bone-repair properties of β-TCP by developing a 3D-printed b-TCP scaffold. Their findings suggested that β-TCP exhibited good biocompatibility and promoted osteogenic differentiation by inducing the expression of osteogenic factors, such as methyltransferase-like 3 (METTL3) and Runx2 [56]. On the other hand, slow biodegradability and extreme fragility limit their use in clinical applications [57,58]. To overcome this problem, ceramic-based composite scaffolds have been developed.
(b) Natural polymers are the most widely used biomaterials due to the high affinity of their structure with the native ECM made up of nano-/microscale protein fibers with different arrangements. They include collagen (Col), alginate (ALG), chitin, and chitosan (CS). Natural polymers have low toxicity, poor immunogenicity, and good biocompatibility, as they are derived from natural sources such as plants, animals, and microorganisms; they also possess the ability to stimulate cell growth and adhesion, thereby promoting bone tissue regeneration [59,60]. Lin et al. showed that natural collagen derived from marine sponge was able to promote cell adhesion and mineralization in vitro [61]. Similarly, Sukul et al. investigated the effect of chitosan sponges on the adhesion, growth, and differentiation of primary human osteoblasts, suggesting that 3D sponges could contribute to angiogenesis and bone remodeling [62]. Nonetheless, low mechanical stability, poor osteoinductivity, and quick biodegradability limit their application compared with other ceramic or metal biomaterials [63]. To overcome these drawbacks, natural polymers have been combined with other materials.
(c) Synthetic polymers, including polystyrene, PLA, PGA, PCL, and polylactic-co-glycolic acid (PLGA), are often used, due to the possibility of regulating their mechanical properties, biodegradability, morphology, and structure during the fabrication process [64,65]. Recently, some in vitro studies have shown that 3D-printed PLA scaffolds are able to promote the adhesion, proliferation, and differentiation of osteoblast cells [66,67]. In another study, the osteoregenerative capability of a porous PLGA (P) scaffold combined with magnesium hydroxide (MH, M), bone-extracellular matrix (bECM, E), and bioactive polydeoxyribonucleotide (PDRN, P) (PMEP scaffold) was evaluated. The authors showed that the developed PMEP scaffold displayed remarkable biological properties in terms of cell adhesion, proliferation, and osteogenic differentiation in vitro [68]. Despite these advantages, some important limitations, such as poor biocompatibility, high toxicity, and reduced bioactivity and osteoconductivity have restricted their application in BTE [69]. These limitations can be overcome by combining synthetic polymers with natural polymers or ceramics.
(d) Metals, such as iron, chromium, stainless steel, titanium, and cobalt alloys, are particularly attractive biomaterials for bone implants, due to their exceptional mechanical properties, which include high elasticity, resistance and ductility, and structural stability [70,71]. Deng and colleagues discovered that 3D-printed Ti6Al4V scaffolds promoted bone formation in vivo, which is strongly influenced by scaffold porosity [72]. Despite this, there are several limitations associated with the use of metal as a scaffold, including a high Young’s modulus, poor degradability, metal ion toxicity, and particle release [73]. To limit these disadvantages, it is possible to improve their chemical structures (e.g., porosity), combine them with other biomaterials, or use biodegradable metals, such as magnesium, zinc, and calcium [74].
(e) Composite (or hybrid) biomaterials are made by combining two or more biomaterials, such as co-polymers, polymeric/ceramic or metallic/ceramic compounds, and metal implants coated with polymers (PLA/PGA, PLA/HA, PGA/PCA, HA/PGA, HA/CS, or HA/Col) [75]. Calabrese et al. showed that hybrid scaffolds made of collagen and hydroxyapatite are able to induce osteogenic differentiation in hADSCs and stimulate bone augmentation after ectopic transplantation in mice [76,77]. Hence, these biomaterials yield improvements in terms of their biological, chemical, and structural properties, although their manufacturing procedures are laborious [78].
(f) Hydrogels are hydrophilic polymers that have a high absorption capacity for water or biological fluids [79]. They are good candidates for BTE applications due to their elastic nature, which is comparable to that of ECM [80,81]. Hydrogels can be of natural (e.g., hyaluronic acid) or synthetic (e.g., polyethylene oxide (PEO)) origin. In this context, a hyaluronic acid-based hydrogel combined with BMP-2 and human MSCs was found to increase cell survival in vitro and to encourage bone formation and vascularization in vivo [82]. Jo et al. demonstrated that the injection of chitosan-PEO hydrogel, in combination with BMP-2 and MSCs, promoted bone formation in vivo [83]. Despite their limitations in terms of biocompatibility and biodegradability in vivo [84], their flexibility, i.e., the ability to adjust the structural parameters during the manufacturing processes [85], and the possibility of minimally invasive implantation [86], strongly encourage their application in the BTE field.

Table 1 summarizes the most widely used biomaterials in BTE applications and their advantages and disadvantages.

Considering the above, there is currently no biomaterial that completely satisfies the desired requirements to promote bone regeneration, particularly regarding mechanical and biological properties. Therefore, in recent years, considerable efforts have been made to develop functional scaffolds by combining biomaterials with nanomaterials in order to obtain ideal substitutes that restore, maintain, or improve damaged tissue.

## 4. Nanotechnology

In the last few years, the application of nanotechnology to BTE has gained great interest. Nanotechnology can offer innovative solutions to improve the mechanical, chemical–physical, and biological properties of scaffolds [87]. Nanomaterials are the key elements of nanotechnology. They are materials with less than 100 nm size in at least one of their dimensions; they include nanoparticles, nanoclusters, nanocrystals, nanotubes, nanofibers, nanowires, nanorods, nanofilms, etc. [88]. Their nanoscale dimensions enhance their chemical–physical properties, giving them unique properties that make them successful in many biomedical applications [89]. Among such nanomaterials, nanoparticles (NPs) have been broadly investigated as potential candidates in the BTE field. NPs offer the possibility of developing biocompatible scaffolds that mimic tissue-specific microenvironments by offering appropriate tensile strength, releasing biological factors, and enhancing cell adhesion, proliferation, and differentiation, thereby promoting tissue growth [87]. Recent research has shown that NPs are able to affect bone regeneration by improving cell signaling, proliferation and osteogenic differentiation [90,91]. NPs used in biomedical applications generally have an average size of between 10 and 100 nm [92]. They can be produced from different types of materials, such as ceramics, natural and synthetic polymers, metals, and organic materials [93,94], and they are generally combined with different matrices to develop nanocomposite scaffolds [95,96].

NPs, based on their chemical composition, can be classified into three categories: organic, inorganic, and carbon-based [97].

## 5. Organic NPs

Organic NPs are made-up of organic materials including lipids, proteins, carbohydrates, and other organic compounds. They include polymeric NPs, liposomes, and dendrimers, that are generally non-toxic and biodegradable, and some have a hollow sphere (i.e., liposomes).

### 5.1. Natural Polymers

Collagen (type I) (Col), the major organic element of bone matrix, is becoming an increasingly essential component of novel implants for BTE applications due to its high biocompatibility and osteoconductivity. Nevertheless, some disadvantages, including weak mechanical properties, rapid degradability, and poor osteoinductivity, still limit its use for orthopedic devices [98]. To overcome these drawbacks, many attempts have been made to improve conventional implants for bone tissue repair using Col NPs. For example, collagen–apatite (Col–Ap) has been shown to promote osteoblast proliferation and differentiation and improve vascularization at the defect site in vivo [99]. Similarly, Col-hydrogel nanocomposites have been found to enhance bone mineralization in vivo [100]. Gresita et al. reported that collagen-coating Hyperelastic Bone (HB), a biocompatible synthetic polymer consisting of 90% HA and 10% PLGA, strongly improved osteoblast adhesion and proliferation of MG-63 human osteosarcoma cells over 7 days of culture in vitro [101].

Gelatine (Gel) obtained from the hydrolysis of collagen is an intriguing natural polymer for nanotechnology applications. Gel NPs have been widely used as drug and gene carriers and could be a valid candidate for novel BTE applications due to their low toxicity and cost-effectiveness, as well as their great bioactivity and biodegradability [102]. In their in vitro study, Loyo et al. reported a synergic effect of gel and graphene oxide (GO) NPs, enhancing the multifunctionality of a polycaprolactone (PCL) nanofibrous scaffold in terms of the degradability rate, bioactivity, and cell adhesion and proliferation of human gingival mesenchymal stem cells (hGMSC cells), making it an interesting biomaterial for BTE applications [103].

Chen et al. developed core-shell nanofibers of HA/Gel-CS to mimic both the structure and chemical composition of native bone and demonstrated that this composite improved osteoblast cell proliferation [104]. Li et al. demonstrated that the integration of gel in fibrous scaffolds of PCL and CaP stimulated adhesion, proliferation, and the mineralization of preosteoblastic MC3T3-E1 cells, suggesting that PCL/HAp/Gel composite fibrous scaffolds could be a good option for bone tissue engineering [105]. In another study, it was demonstrated that 3D nanocomposites based on gold nanoparticles (Au NPs) and gel nanofibers promoted bone regeneration both in vitro and in vivo by mimicking the natural bone structure [106].

Fibrin, a natural biopolymer involved in the coagulation process, provides support for the synthesis of ECM due to its nano-scaffold nature, promoting cell adhesion and proliferation during wound healing and bone growth [107]. Their precursors (fibrinogen and thrombin) can be obtained from a patient’s blood, allowing the development of purely autologous and low-cost scaffolds which could be controlled during the manufacturing process by adjusting the component concentrations. Kim and Lee investigated the effect of a fibrinogen coating on the surface of biphasic calcium phosphate (BCP) in vitro and reported that it markedly improved human mesenchymal stem cell (hMSC) proliferation and adhesion. Furthermore, it was shown that in vivo implantation of BCP granules coated with fibrinogen significantly enhanced bone healing [108]. Likewise, Santos et al. noted that embedding fibrinogen into CS scaffolds enhanced bone regeneration in vivo [109]. Another benefit is the possibility of injecting fibrin as a liquid that solidifies in situ, representing a minimally invasive procedure. However, because of its rapid degradation and weak mechanical properties [110], many researchers have combined fibrin with nanotechnology to overcome these limitations [111]. In this regard, Periyathambi et al. developed magnetic fibrin NPs which were able to enhance cell viability and the ALP activity of Saos-2 cells in vitro [112].

Alginate (ALG) is a polymer that is abundantly found in the cell walls of a variety of brown algae species [113]. Because of its low toxicity and non-immunogenicity, as well as its good biocompatibility, bio-adhesiveness, and biodegradability, researchers have focused their interest on its use for biomedical applications, including drug delivery and TE strategies [114,115]. In this regard, several studies have demonstrated that HA/ALG nanocomposites exhibit enhancements in terms of cell adhesion, proliferation, and osteogenic differentiation when compared with a pure ALG scaffold [116,117,118].

### 5.2. Synthetic Polymers

PLA has gained attention, since it has a simple manufacturing processes and a good degradation rate, comparable to the healing process of damaged human bone tissue, even if its mechanical and biological properties leave room for improvement [119]. Thus, scientists are actively exploring the possibility of employing PLA nanofibers for innovative BTE strategies. Several studies have demonstrated the ability of PLA nanofibers, used alone or in combination with other molecules, to promote cell growth and osteogenic differentiation in vitro [120,121,122]. Liu et al. developed a PLA nanocomposite fiber mat with GO and nanohydroxyapatite (nHA) and demonstrated that the nanocomposite scaffold exhibited high biocompatibility, tensile strength, and modulus, as well as excellent cell proliferation [123].

PLGA, a synthetic copolymer containing lactic and glycolic acid at different ratios, has received significant interest due to its excellent biocompatibility, efficient biodegradability, and manipulable mechanical properties [124] as a drug delivery system for the treatment of large bone defects [125]. In this regard, several studies have shown how PLGA-based nanomaterials can enhance bone formation in vitro and in vivo due to their ability to deliver growth factors, including BMP-2 [126,127,128]. Tian et al. developed a new UPPE scaffold, named UPPE-PLGA-rhBMP-2, by incorporating PLGA microspheres containing recombinant human (rh) BMP-2 to improve its osteoinduction properties. They showed that the newly developed scaffold enhanced the ALP activity of bone marrow stromal cells (bMSCs) cultured on them, indicating that the incorporation of PLGA-rhBMP-2 increased the osteoinductive properties compared to the native UPPE scaffold [129].

### 5.3. Liposomes

Lipid NPs are one of the most widely used drug delivery systems due to their exceptional biocompatibility, ease of drug release, and passive targeting ability. Nevertheless, many disadvantages limit their use in clinical settings, especially in bone regeneration, including difficulties in transporting, storing, and maintaining the concentration of the drug in situ. Liposomes are lipid structures at the nanoscale (25 nm–2.5 μm), consisting of an amphipathic bilayer primarily composed of phospholipids and cholesterol that surrounds a hydrophilic core [130]. They can transport drugs directly in situ and maintain them there for a long time without causing damage [131]. However, conventional liposomes are not naturally able to promote bone regeneration, because they typically contain significant concentrations of non-bioactive lipids such as cholesterol and phospholipids. Therefore, recently, an innovative osteoinductive liposomal formulation containing oxysterols was developed to improve the properties of standard liposomal formulations [132]. In recent years, different types of scaffolds integrating liposomes have been developed to combine a healing effect with mechanical support for more efficient bone regeneration. Cheng et al. demonstrated that loading liposomes into BTE scaffolds aided in the solubilization and stabilization of bioactive cargo, improving bioavailability and retention [133]. Wang et al. developed a drug delivery system by combining composite scaffolds made up of collagen and hydroxyapatite (Col/HA) with bisphosphonate (BP)-derivatized liposomes to provide a sustained drug release platform in bone regeneration and repair [134]. Lee et al. developed an innovative liposomal delivery system by immobilizing agonists of Hedgehog (HH) signaling (Smoothened agonist (SAG)) onto apatite-coated 3D scaffolds to enhance Hh signaling and, therefore, bone healing. Their results showed a substantial and dose-dependent increase in Hh-mediated osteogenic differentiation in vitro and improved bone repair in vivo [135]. In addition, the osteogenic potential was improved when this approach used to deliver osteogenic molecules, including purmorphamine, smoothened agonist (SAG), and signaling molecule sonic hedgehog (Shh) [136]. Similarly, Cottrill et al. reported that the integration of 20S-hydroxycholesterol and stearyl amine (SA) non-phospholipid liposomes in a methacrylate glycol chitosan (MeGC) hydrogel scaffold effectively stimulated osteogenesis in vitro and bone healing in vivo [137].

### 5.4. Dendrimers

Dendrimers are nano-sized symmetric molecules consisting of tree-like arms or branches called dendrons [138]. These NPs are able to improve the surface chemical–electrical properties, as well as biodegradability, and mimic natural ECM, thus allowing for novel uses in TE [139]. Kurian et al. developed a multifunctional composite hydrogel consisting of photo-responsive Gelatin Methacryloyl (GelMA) and dendrimer (G3)-functionalized nanoceria (G3@nCe/GelMA) and reported that it improved cell adhesion, proliferation, and osteogenic differentiation of MSCs in vitro [140]. Furthermore, they demonstrated that when implanted subcutaneously, G3@nCe/GelMA hydrogel exhibited excellent tissue integration and minimal inflammatory response. Dendrimers were also investigated in vitro as gene delivery vectors to improve osteogenic differentiation by carrying a BMP-2 gene-containing plasmid. In their study, polyamidoamine (PAMAM) dendrimers carrying the hBMP-2 gene (PAMAM/hBMP-2) were used for the transfection of MSCs to promote osteogenic differentiation in vitro. The results obtained indicated that the PAMAM/hBMP-2 system was able to strongly stimulate the osteogenic differentiation of MSCs in vitro [141]. Oliveira et al. developed carboxymethylchitosan/poly(amidoamine)dendrimer nanoparticles (Dex-loaded CMCht/PAMAM NPs) as carriers to deliver bioactive molecules aimed at inducing the osteogenic differentiation of rat bone marrow stem cells [142]. In recent studies, the same authors showed that a combination of hydroxyapatite (HAp) scaffolds, bone marrow stromal cells, and Dex-loaded CMCht/PAMAM dendrimer enhanced osteogenesis in vitro (3-D systems) and de novo bone formation in vivo [143].

## 6. Inorganic NPs

Inorganic NPs include NPs that are not made of carbon or organic materials, such as metal, ceramic, and magnetic NPs. These are generally non-toxic, biocompatible, and hydrophilic [144].

### 6.1. Metal NPs

Metal NPs and their oxides have attracted great interest due to their distinctive features, such as mechanical strength, antimicrobial activity, osteogenic and angiogenic potential, and photosensitive properties [145]. Several studies have reported that the chemico–physical properties of metal and metal oxide NPs, such as chemical composition, size, shape, and surface chemistry, can significantly affect their toxicity in biological environments [146,147]. This is mainly because the size and surface area of NPs range from 2 to 10 nm, so they can easily pass cell barriers and enter cell organelles, damaging them. De Jong et al., in their in vivo study, showed that the distribution of gold NPs in organs was highly size-dependent. Specifically, they demonstrated that NPs smaller than 10 nm were found in a greater number of organs than larger NPs [148]. Huo et al. showed that 6-nm NPs can freely enter the cell nucleus, while 10–16-nm NPs can only be found in cytoplasm and cell membranes; thus, gold NPs with less than 10-nm size exhibited higher toxicity [149]. Furthermore, it is known that round-shaped NPs are more susceptible to endocytosis, while plate-like and needle-like NPs cause greater physical damage to cells and live tissue by direct contact than NPs with other geometries [150,151]. In addition to shape and size, the chemical composition must also be considered. Some studies have reported that NPs with similar shape and size but distinct chemical composition have different toxic activities. Yang et al. demonstrated that SiO_2_ and ZnO NPs with the same sizes exhibited different toxicities, reporting that SiO_2_ caused oxidative stress while ZnO induced DNA damage, mainly due to the loss of metal ions in cells [152]. Nevertheless, despite this evidence, it has now been found that when the proper sizes and amounts are used, such NPs are extremely advantageous in biomedical applications [153]. Furthermore, metal and metal oxide NPs possess various advantages, including the possibility of producing NPs of the required size and shape, large surface area, high stability and bioavailability, easy functionalization, and integration into hydrophilic and hydrophobic systems [154]. In recent years, different types of metals and metal oxide NPs, such as gold (Au), silver (Ag), palladium (Pd), titanium (Ti), copper (Cu), zinc oxide (ZnO), titanium dioxide (TiO_2_), and copper oxide (CuO), have been examined in the field of bone regeneration [96,155,156].

Silver nanoparticles (Ag NPs) are widely used in the orthopedic field due to their antibacterial, antifungal, antiviral, anti-inflammatory, and osteoinductive effects, as well as their ability to improve wound healing [157,158]. In the BTE field, Ag NPs have been used to develop nanocomposite scaffolds with dual functions, i.e., antibacterial and osteogenic, to efficiently minimize the risk of microbial infection and inflammation and to promote bone regeneration and wound healing [159]. Hasan et al. developed a nanocomposite of CS, carboxymethyl cellulose and Ag NP-modified cellulose nano whiskers (CS/CMC/CCNWs- Ag NPs) with the ability to provide mechanical strength and antimicrobial activity. They tested the antibacterial activity of the nanocomposite scaffold against Gram-negative and Gram-positive bacteria, compared with an Ag-free scaffold, showing 100% antibacterial efficiency for the Ag-coated scaffold [160]. It is also known that Ag NPs stimulate cell proliferation, differentiation, and the mineralization of osteoprogenitor cells [161]. Zhang et al., in an in vivo study, showed that Ag NPs efficiently promoted MSC proliferation and differentiation toward osteoblasts, thus improving bone fracture healing [162]. Furthermore, Ag NPs are often used as coatings of metallic scaffolds and implants to promote bone regeneration and exert broad-spectrum antibacterial effects, thereby reducing implant-associated infection risk [163,164,165].

Gold nanoparticles (Au NPs) have recently gained interest in different biomedical applications, such as drug delivery, cell targeting, biosensing, and TE, due to their good biocompatibility, photothermal stability, facile synthetic method, and versatile surface functionalization [166]. Specifically, Au NPs have been designed as favorable candidates for bone regeneration. It has been reported that Au NPs are able to promote the osteogenic differentiation of MSCs [167,168] and the mineralization of primary osteoblasts [169], to inhibit osteoclast differentiation and bone resorption [170], and to improve bone regeneration in both in vitro and in vivo models [171,172]. Furthermore, it has been shown that the biological functions of Au NPs are influenced by their size, concentration, and surface chemistry [173,174], as well as the osteogenic differentiation of MSCs [175]. In their study, Li et al. investigated the effects of Au NPs with different shapes and diameters and demonstrated that the osteogenic differentiation of hMSCs was dependent on their size and shape. Specifically, they showed that 70-nm, rod-shaped Au NPs significantly increased the osteogenic differentiation of hMSCs in contrast to 40-nm, rod-shaped Au NPs, which suppressed it [176]. In another study, 30- and 50-nm sized spherical Au NPs were reported to be the most effective at promoting osteogenic differentiation of ADSCs compared to 75- and 100-nm sizes [177].

Palladium NPs (Pd NPs) showed great potential in biomedical applications due to their exceptional physicochemical properties, such as great thermal and chemical stability, significant photocatalytic activity, electronic, and optical properties, and low cost [178]. Recently, Pd NPs have been used as photothermal agents [179], photoacoustic agents [180], anticancer agents [181], antimicrobial agents [182], gene/drug carriers [183], and prodrug activators [184]. Pd NPs have also been applied to nanocomposite scaffolds to enhance their physical–chemical and biological properties. Ismail et al. developed an innovative polyvinyl alcohol/ALG (PVA/Alg) composite scaffold loaded with green-synthesized Pd NPs. Their results suggested the loading with Pd NPs provides an appropriate mechanical support, increases cell viability, and produces an extracellular and mineralized matrix [185]. Murugesan et al. investigated the effects of Pd NPs on nanocomposite scaffolds composed of reduced graphene oxide (rGO) functionalized with polypyrrole (PPy) (Pd/PPy/rGO). Their results showed that Pd NPs prevented colonization, adhesion, and biofilm formation on scaffold surfaces [186]. Despite this evidence, other studies reported cytotoxic effects of Pd NPs. Calabrese et al. reported that Mg-HA-Col type I scaffolds functionalized with Pd NPs inhibited cell growth and decreased cell differentiation [90].

Copper (Cu) is an essential mineral that is involved in many biological processes, including bone metabolism regulation and the formation and maintenance of myelin [187]. In recent years, Cu^2+^ ions have gained great interest in the BTE field due to their unique features, such as antibacterial properties [188], anti-inflammatory activity, ability to stimulate angiogenesis and collagen deposition [189], and ability to induce osteogenic differentiation of MSCs [190]. Tripathi et al. developed CS/nHA bio-composite scaffolds containing Cu–Zn NPs (CS/nHAp/nCu–Zn) in order to improve their antibacterial and osteoproliferative properties, thereby minimizing the risk of implant-associated bacterial infection and promoting bone formation [191]. In another study, Vilardell et al. showed that the addition of 3 at.% Cu to Ti6Al4V(ELI) alloyed materials inhibited the attachment of *S. aureus* and *E. coli* and decreased biofilm formation [192]. Wu and colleagues reported that a Cu-containing mesoporous bioactive glass (Cu-MBG) scaffolds significantly promoted the osteogenic differentiation of hBMSCs, inhibited bacteria viability, and enhanced angiogenesis, indicating that Cu^2+^ ions offered multifunctional properties to MBG scaffolds [193]. In another study, Ewald et al. reported that Cu^2+^ ions enhanced the cell activity and proliferation of osteoblastic cells seeded on brushite (CaHPO(_4_) · 2 H(_2_) O) scaffolds; furthermore, Cu^2+^ ions were found to affect the expression of many bone-related proteins, including bone sialoprotein and osteocalcin [194]. Copper oxide (CuO) NPs are widely used in nanomedical applications due to their strong bactericidal, catalytic, anti-carcinogenic, and coating activities [195]. Sahmani et al. used CuO NPs to improve the mechanical properties, cell viability, and electrical conductivity of a n-HA scaffold [196].

Zinc is involved in many physiological processes and plays a key regulatory role in osteogenesis and in bone homeostasis [197,198]. Zinc oxide NPs (ZnO NPs) are the most common type of zinc-containing nanoparticles; they have received great attention in many biological fields due to their low toxicity, good biocompatibility, high antibacterial and anticancer activities, and better osteogenic properties, being able to promote bone growth and mineralization [199,200,201]. The above properties have made ZnO NPs promising candidates in orthopedic applications. Numerous studies have recently investigated the possibility of using ZnO NPs as doping or coating agents for BTE implants to improve their antibacterial and osteogenic properties [202,203]. In this regard, Shen et al. incorporated a series of ZnO NPs on microrough titanium (Ti) to increase the biological functions of a Ti implant. They demonstrated that Ti-ZnO scaffolds effectively inhibited bacterial adhesion and were able to regulate the proliferation and differentiation of osteoblasts and osteoclasts in vitro and, more importantly, promote new bone formation in vivo [204]. In another study, biomimetic nanofibrous scaffolds of PCL/nHA were electrospun with different concentrations of ZnO NPs (1wt%, 5wt%, 10wt%, 15wt% and 30wt%) to evaluate the optimal range of NPs with good biocompatibility and osteoregenerative activity. Their results showed that although PCL/nHA/ZnO scaffolds with higher concentrations of ZnO NPs exhibited superior antimicrobial efficacy, a significant decrease in cell viability and mechanical properties was observed. Therefore, PCL/nHA/ZnO scaffolds with 10wt% ZnO showed optimal cell viability, antimicrobial effects, and mechanical strength [205]. In the study of Maimaiti et al., HA NPs and ZnO NPs were uniformly coated on the surface of a Ti substrate. Their results showed that a HA/Zn coating yielded stronger antibacterial and osteoinductive effects compared to a pure HA coating [203]. Calabrese et al, developed nano-functionalized Ti scaffolds with colloidal ZnO NPs and Mn-doped ZnO NPs (ZnO@Ti and Zn_x_Mn_(1−x)_O@Ti) exhibiting higher antibacterial activity than a pure Ti scaffold [206].

Ti and its alloys are the most widely used metals for joint replacement due to their excellent properties, such as high strength, good biocompatibility, extreme corrosion resistance, and good bone affinity [207]. However, even if Ti possesses these excellent properties, smooth-surface Ti implants lack good osseointegration capacity and antibacterial activity, often resulting in implant failure. Therefore, several studies have been focused on improving these properties through surface modifications of pure Ti implants [208,209,210]. Ramires et al. reported that TiO_2_/HA coatings onto a Ti substrate promoted cell proliferation and osteogenic differentiation [211]. Calabrese et al., in an in vitro study, observed that Ti scaffolds nano-functionalized with TiO_2_ (Ti_TiO_2_) and γFe_2_O_3_ (Ti_γFe_2_O_3_) exhibited higher antibacterial activity and increased cell proliferation and differentiation, suggesting that nano-functionalized Ti substrates could represent promising prototypes for BTE applications [153]. Similarly, Pan et al. demonstrated that a micro-/nano-hierarchical structured TiO*_2_* coating on a Ti surface significantly increased hydrophilicity, as well as promoting adhesion and osteogenic differentiation in vitro [212].

### 6.2. Ceramic NPs

Ceramic NPs are the most widely used inorganic NPs for bone grafting applications due to their similarity with the inorganic matrix of bone tissue, which allows them to promote osteogenesis [213]. Among these, HA NPs are the most widely used to improve the mechanical and biological properties of several biomaterials. Specifically, HA NPs are generally combined with synthetic polymers like PLA [214,215] and PCL [216,217] in order to overcome their limits for clinical uses by enhancing cell adhesion and mineral deposition in vitro. For the same reason, HA NPs were also used to improve the mechanical and biological properties, as well as the stability under physiological conditions, of hydrogel scaffolds, such as Gellan Gum, in which it was observed that the integration of HA NPs enhanced cell proliferation and ALP activity in vitro [84].

b-TCP is another type of calcium phosphate that has been widely investigated for its potential use in BTE applications. Although b-TCP appears to be less stable than HA, it shows excellent osteoconductivity and osteoinductivity due to its rapid degradation rate and solubility, as well as its nanoporous structure [218]. In this context, it has been shown that applying a b-TCP NP coating to 3D collagen scaffolds enhances cell proliferation and bone formation in vivo [219]. Other studies have demonstrated that the incorporation of b-TCP NPs in conventional 3D BTE scaffolds provides them with a controlled degradation rate and strengthened mechanical properties, also improving in vitro and in vivo biological responses in terms of cell adhesion, viability, and mineralization [220,221].

BCP is made up of a mixture of two CaP phases, i.e., HA (more stable) and b-TCP (more soluble), at different ratios. This combination offers notable advantages compared to the aforementioned CaP bioceramics alone by allowing greater control of bioactivity, biodegradation, and osteoconductivity [222]. In this regard, Nie et al. observed that the incorporation of BCP NPs in CS/Gel hydrogels improved their chemical–physical features, degradation rate, and biocompatibility in vitro. Additionally, in vivo, new bone formation into the scaffolds was observed [223]. In another study, collagen and dexamethasone (DEX)-releasing BCP NPs composite scaffolds showed good porosity, strength, biocompatibility, and osteoinductivity in vitro. Further in vivo findings confirmed that the presence of DEX-loaded BCP NPs facilitated bone tissue repair [224].

There is increasing interest in the application of silica-based materials in BTE due to their important biological function in bone formation by stimulating the synthesis of Col I and osteogenic differentiation [225]. In this regard, mesoporous silica nanoparticles (MSNs) have been deeply investigated for their ability to improve native scaffold features, due to their non-toxicity, high biocompatibility, and adjustable porosity [226]. In particular, MSNs were found to suppress osteoclast resorption, and enhance bone formation and mineralization in vivo [227,228]. Additionally, it has been reported that the coating of HA loaded with MSNs on Ti implants provides excellent biocompatibility and promotes osteoblast differentiation in vitro [229].

### 6.3. Magnetic NPs

Magnetic nanoparticles (MNPs) are composed of metals such as iron or cobalt, endowed with magnetic, semiconductor, biocompatible, and bioactive properties that play an important role in bone regeneration [230,231]. Among these, MNPs superparamagnetic iron oxide nanoparticles (SPIONs), such as magnetite (Fe_3_O_4_) and maghemite (γ-Fe_2_O_3_), have gained particular interest, due to their multifunctional features, such as considerable magnetic, chemical, thermal, and mechanical properties, as well as intrinsic biocompatibility [232,233]. In this regard, in recent years, many studies have examined the effects of SPION integration into scaffolds for bone regeneration. An in vitro study reported that paramagnetic nanofibrous composite films consisting of PLA, HA, and γ-Fe_2_O_3_ NPs improved the proliferation, differentiation, and ECM secretion of osteoblast cells under a static magnetic field [234]. The same authors, in another in vivo study, reported that a nanofibrous composite scaffold consisting of super-paramagnetic γ-Fe_2_O_3_ NPs, HA NPs, and PLA, under a static magnetic field, accelerated bone regeneration in vivo [235]. Singh et al. developed magnetic nanofibrous scaffolds of PCL, integrating MNPs to improve their physical–chemical, mechanical, and biological properties. They showed that MNP incorporation greatly improved the hydrophilicity and tensile mechanical properties of the nanofibers, as well as the degradation rate and mineralization in vitro. Furthermore, the new magnetic nanofibrous scaffolds exhibited improved osteogenesis in vitro and bone regeneration in vivo [236]. Zhao et al. incorporated nHA and Fe_3_O_4_ NPs into a CS/Col organic matrix, showing that CS/Col/Fe_3_O_4_/nHAP magnetic scaffolds possessed superior structural and mechanical performance for cell adhesion and proliferation, as well as osteogenic differentiation in vitro. Furthermore, they demonstrated that the magnetic hybrid micro/nanostructured composite scaffolds improved mineralization and bone regeneration in vivo [237]. Xia et al. developed new, iron oxide nanoparticle-incorporating calcium phosphate cement scaffolds (IONP-CPC) to evaluate their osteogenic activity on human dental pulp stem cells (hDPSCs). They demonstrated that the novel CPC functionalized with IONPs markedly improved cell attachment, osteogenic differentiation, and bone mineralization in the seeded cells [238]. It has also been demonstrated that the integration of MNPs within coatings of HA increases the wettability and corrosion resistance of Ti-based biomaterials, as well as providing better mineralization, cell viability, and proliferation [239].

## 7. Carbon-Based Nanomaterials

Carbon nanomaterials (CNMs) are a class of natural or artificial materials mainly composed of carbon with at least one dimension in the nanoscale. Research on innovative BTE strategies has focused its attention on CNMs due to their superior mechanical strength, stability, adaptable biodegradability, and cost-effectiveness, as well as remarkable biocompatibility and osteoinductive potential. Depending on their structure and size, each class of CNM exhibits specific properties and functions. In light of this, they are mainly classified as: (a) zero-dimensional (0D) CNMs, including fullerene, nanodiamonds (NDs), and carbon dots (CDs); (b) one-dimensional (1D) carbon nanotubes (CNTs); (c) two-dimensional (2D) graphene and its derivatives; and (d) three-dimensional (3D) CNMs, such as graphite and diamond, although these have not been extensively investigated for BTE applications due to their lack of pores, which represent an important requirement for cell adhesion, growth, and differentiation [240,241].

### 7.1. 0D CNMs

0D CNMs are mainly spherical or quasi-spherical NPs that are characterized by high surface-to-volume ratios and ultra-small sizes, which make them exceptionally suitable for use in biomedical applications [242].

One of the most widely studied 0D CNMs is fullerene (C60), which is generally composed of sixty carbon atoms arranged in a spherical shape as a result of encircling a single sheet of graphene [243]. Several studies have demonstrated that fullerene, especially in the form of continuous and micropatterned films, promotes osteoblast adhesion, proliferation, and differentiation [244,245,246], although it was found to exhibit low solubility in physiological fluids [240], as well as considerable cytotoxicity [247]. To overcome these limits, fullerene is generally functionalized with a wide range of polymers (i.e., PEG) [248,249,250].

NDs have also attracted a lot of interest among 0D CNMs for BTE applications. With a size of ∼5 nm and a large surface/volume ratio, NDs exhibit great surface reactivity, as well as extreme hardness, chemical stability, and biocompatibility. They are generally functionalized or oxidated in order to overcome their hydrophilicity and dispersion limits [251]. In particular, ND films were found to be promising for BTE applications due to their ability to promote in vitro osteoblast adhesion, proliferation, and differentiation [252]. Furthermore, it has been demonstrated that the incorporation of NDs in conventional BTE scaffolds, such as PLA and PGLA, provided an enhancement of their mechanical and biological properties [253,254].

In recent years, CDs have received great interest in many biomedical applications due to their specific features, such as strong fluorescence, tunable surface properties, high water solubility, and low cytotoxic potential [255]. Several studies have recently focused on their potential use for BTE applications. Khajuria et al. developed an innovative scaffold combining nitrogen-doped carbon dots (NCDs) with HA, demonstrating its osteogenic activity in vitro and in vivo [256]. In vitro studies have demonstrated that the integration of CDs into composite materials improves mechanical strength, as well as cell adhesion and proliferation [257,258]. In addition, in vivo studies showed that CDs exhibit low cytotoxicity and high biocompatibility, suggesting their potential use for BTE approaches [259,260]. In this regard, in their study, Gogoi et al. reported that the combination of CDs with HA scaffolds promotes osteoblast proliferation and mineralization both in vitro and in vivo [258].

### 7.2. 1D CNMs

CNTs are the most common representation of 1D CNMs and are usually divided into two categories: single-walled carbon nanotubes (SWCNTs) and multi-walled carbon nanotubes (MWCNTs) [261]. Due to their unique properties, which include excellent mechanical, electrical, and thermal properties, as well as easy surface modification, high biocompatibility, and non-immunogenicity, CNTs have captured the interest of researchers for potential use in new BTE strategies [262,263]. In this context, it has been demonstrated that MWCNTs can enhance BMP-2, ALP, and Collagen I expression, thereby promoting osteogenic differentiation in vitro and ectopic bone formation in vivo [264]. However, surface functionalization with other biomaterials is generally adopted to improve their biocompatibility and dispersion [265]. On the other hand, functionalized CNTs have shown great potential in enhancing the mechanical and biological properties of several conventional BTE scaffolds, including HA [266,267], collagen [268], PLA [269,270], PLGA [271,272,273], and PCL [274,275].

### 7.3. 2D CNMs

Graphene and its derivatives, i.e., GO and reduced rGO, represent the 2D CNM group. With distinct mechanical and electrical properties, as well as high surface area and chemical stability, graphene and its derivatives represent valid candidates for BTE approaches [276]. Recently, it was reported that graphene enhances cell adhesion, proliferation, and osteogenic differentiation [277,278], and that it can be used in combination with HA to develop improved scaffolds for bone regeneration [279,280]. Among graphene derivatives, GO represents the most attractive alternative for BTE approaches, since it provides more active sites for the surface functionalization of pure biomaterials in order to improve their mechanical and biological properties [281,282,283].

Despite the fact that rGO has shown decreased chemical versatility, hydrophilicity, and dispersion [284], it also exhibits a good mechanical strength and osteoinductivity, which make it a potential candidate for scaffold development [285]. Lu et al. designed novel rGO hydrogels for BTE applications and found that the integration of rGO improved biocompatibility and osteoinductivity, both in vitro and in vivo [286,287]. Furthermore, some studies have reported that the combination of rGO with conventional BTE scaffolds, especially HA-based ones, enhanced bone growth and mineralization, both in vitro and in vivo [288,289,290].

Table 2 reports the different types of NPs and the effects of their integration into biomaterials for BTE applications.

## 8. Conclusions

Restoring large bone defects is still a big challenge in the orthopedic field due to a lack of treatments which are able to satisfy all the clinical needs. Therefore, it is essential to evaluate new therapeutic approaches that can improve the quality of life of patients, avoiding side effects, including pain, donor site morbidity, rejection, transmission of diseases, and high cost. In recent years, the BTE strategy has gained great attention, but although considerable efforts have been made to develop ideal biomaterials that are capable of satisfying all clinical needs, to date, this technology is still not able to address all the complications of conventional approaches. In this context, the use of nanotechnology in the field of BTE has emerged as a promising approach for the development of more efficient biomaterials due to the possibility engineering and manipulating materials on the nanoscale. In this review, we have discussed the effects of the integration of different types of organic, inorganic, and carbon-based NPs on both bone cells and biomaterials for BTE. Specifically, we have reported on some in vitro and in vivo studies that highlighted how the integration of NPs in biomaterials can improve both the properties of the scaffolds themselves and the behavior of the cells grown on them, in terms of cell proliferation and differentiation. However, although the reported evidence suggests that the combination of nanotechnology with BTE could represent an innovative tool for the treatment of large bone defects, further preclinical investigations will be needed to evaluate their long-term effects in biological systems. Therefore, future efforts in this area will be made to overcome the toxicity and immunogenicity problems associated with the release and biodegradation of NPs, as well as to improve manufacturing processes to develop more efficient and cost-effective biomaterials. Furthermore, it will be necessary to investigate the molecular mechanisms that underlie the interactions between cells and nanofunctionalized biomaterials in order to activate the signaling pathways involved in bone tissue regeneration and repair. Together, the studies described in this review offer important information that could contribute to the development of an innovative approach for the treatment of large bone defects, which currently have a major impact on global health.

## Figures and Tables

**Table 1:** Advantages and disadvantages of biomaterials employed for the BTE applications.

Class of Biomaterial	e.g.,	Advantages	Disadvantages	References
Ceramics	Bioglass, alumina, zirconia, CaP (HA, b-TCP, biphasic calcium phosphate)	Biocompatibility, osteoconductivity.	Slow degradation, shaping issues, fragility.	[48,49,50,51,52,53,54,55,56,57,58]
Polymers	Naturals (Col, ALG, chitin, CS)	Biocompatibility, bioactivity.	Immunogenicity due to pathogenic contaminants, quick biodegradability, weak mechanical qualities.	[59,60,61,62,63]
	Synthetics (polystyrene, PLA, PGA, PCL, PLGA)	Possibility to customize the synthesis procedure and reagents.	Poor mechanical resistance and biocompatibility, toxicity due to the release of ions and other residual particles.	[64,65,66,67,68,69]
Metals	Iron; chromium; stainless steel; titanium- and cobalt-alloys.	High elasticity, resistance, and ductility.	Toxic ion release.	[70,71,72,73,74]
Composites	PLA/PGA; PLA/HA; PGA/PCA; HA/PGA; HA/CS; HA/Col	Improvement of biological, chemical, and structural properties compared to individual components.	Laborious manufacturing procedure.	[75,76,77,78]
Hydrogels	Naturals (e.g., hyaluronic acid) or synthetic (e.g., PEO)	Rubbery ECM-like nature; flexibility to adjust structural parameters during the production process, minimally invasive implant.	Poor biocompatibility and rapid biodegradability in vivo.	[79,80,81,82,83,84,85,86]

**Table 2:** NPs for BTE applications.

Class of NPs	NP Composition	Composite Scaffold	Model In Vitro/In Vivo	Effect of NPs Integration on Scaffold	References
**ORGANIC**					
**Natural** **Polymers**	Collagen	Col-Ap	Rodent	Promotes osteoblast proliferation, differentiation, and vascularization;	[99]
		Col-hydrogel	SBF, mouse	Enhances bone mineralization	[100]
		Collagen-coated HB	MG-63	Improves osteoblast adhesion and proliferation in vitro	[101]
	Gelatin	Gt-coated PCL/GO	hGMSCs	Enhances degradability rate, bioactivity, and cell adhesion and proliferation	[103]
		HA-Gel-CS	Osteoblast cells	Improves cell proliferation	[104]
		3D Gel-Au NPs	MG-63, rat	Promotes bone regeneration	[106]
	Fibrin	Fibrinogen-BCP	hMSCs, rabbit	Promotes cell proliferation and adhesion, as well as bone healing	[108]
		Fibrinogen-CS	Rat	Enhances bone regeneration	[109]
		Magnetic fibrin NPs	Saos-2	Enhances cell viability and ALP activity	[112]
	Alginate	HA/ALG	human osteoblastic cells, rat	Enhances cell adhesion, proliferation, and osteogenic differentiation	[116,117,118]
**Synthetic** **Polymers**	PLA	-	hMSCs	Promotes growth and osteogenic differentiation	[120,121,122]
		PLA-GO-nHA	Saos-2	Enhances biocompatibility, tensile strength, and cell proliferation	[123]
	PLGA	UPPE-PLGA-rhBMP-2	bMSCs	Improves osteogenic differentiation and ALP activity	[129]
**Liposome**	-	20S-OHC/PA/HA	bMSCs, mouse	Improves osteogenesis and bone healing	[135]
	-	20S-OHC-SA-MeGC hydrogel	MSCs, Rat	Stimulates osteogenesis and bone healing	[137]
**Dendrimers**	-	G3@nCe/GelMA hydrogel	rMSCs, rat	Improves cell adhesion, proliferation, osteogenic differentiation, and tissue integration	[140]
	-	PAMAM/hBMP-2	MSCs	Promotes osteogenic differentiation	[141]
**INORGANIC**					
**Metals**	Ag	CS/CMC/CCNWs-AgNPs	MG63 cells, gram (−) and gram (+) bacteria	Promotes mechanical strength, antimicrobial activity, and cell adhesion and proliferation	[159]
		-	Mouse	Promotes osteoblast proliferation and differentiation	[160]
		BMP/CS/Ag/HA-Ti	Osteoblasts, bMSCs, Rabbit, *S. epidermidis* and *E. coli*.	Improves osteoinductivity, bone formation, and antibacterial properties	[163]
	Au	-	MSCs, hADMSCs	Promotes osteogenic differentiation	[168,169]
		-	Primary osteoblasts	Stimulates differentiation and mineralization	[170]
		GNPs-ALD	Bone marrow- derived macrophage, mouse	Inhibits osteoclast differentiation and bone resorption	[171]
		Gel-GNP	ADSCs, rabbit	Improves bone regeneration	[172]
		PEGylated GNPs	MC3T3-E1, hBMSCs, rBMSCs, rabbit	Improves osteogenic differentiation and bone regeneration	[173]
	Pd	PVA/Alg/Pd	hDPSCs	Improves mechanical support and increases cell viability and matrix mineralization	[185]
		Pd/PPy/rGO	*E. coli*, *B. subtilis*, *P. aeruginosa*, and *K. pneumoniae*	Prevents colonization, adhesion, and biofilm formation	[186]
		Mg-HA-ColI-Pd	hADSCs	Inhibits cell growth and decreases cell differentiation	[90]
	Cu, CuO	CS/nHAp/nCu–Zn	rat osteoprogenitor cells	Enhances antibacterial and osteoproliferative properties	[191]
		Ti6Al4V(ELI)-3at.%Cu	hOB, *S. aureus* and *E. coli*	Unaffected by osteoblast behaviour, reduces bacterial adhesion and biofilm formation	[192]
		Cu-MBG	hBMSCs	Promotes osteogenic differentiation, inhibits bacteria viability, and enhances angiogenesis	[193]
		n-HA-CuO	In vitro	Improves mechanical properties, cell viability, and electrical conductivity	[196]
	ZnO	HA/PPy/ZnO/Ti	* E. coli * and *S. aureus*, bMSCs.	Promotes antibacterial and osteoinductive activity	[203]
		Ti-ZnO	* S. aureus * and *P. aeruginosa*, Osteoblast and osteoclast cells, rabbit	Inhibits bacterial adhesion, regulates the proliferation and differentiation of osteoblasts and osteoclasts, and promotes new bone formation	[204]
		PCL/nHA/ZnO	MG-63 cells, E. coli and *S. aureus*	Improves cell viability, antimicrobial effect, and mechanical strength	[205]
		ZnO@Ti and Zn_x_Mn_(1−x)_O@Ti	* S. aureus * and *P. aeruginosa*	Exhibits antibacterial activity	[206]
	TiO_2_	TiO_2_/HA/Ti	Osteoblast cells	Promotes cell proliferation and osteogenic differentiation	[211]
		Ti_TiO_2_	*S. aureus*, hADSCs	Promotes anti-bacterial activity, cell proliferation and differentiation.	[153]
		Ti_TiO2	MG63 cells	Promotes adhesion and osteogenic differentiation	[212]
**Ceramics**	HA	PLA-HA PCL-HA	hADMSCs, osteoblasts, rat	Enhances cell adhesion and mineral deposition	[214,215,216,217]
		Gellan Gum/HA	7F2 osteoblast cells	Enhances cell proliferation and ALP activity	[84]
	b-TCP	3D collagen-b-TCP	Osteoblast cells rat	Enhances cell proliferation and bone formation	[219]
		PCL-b-TCP	rat bone mesenchymal stem cells, rat	Enhances cell adhesion, viability, and mineralization, and promotes bone formation	[220,221]
	BCP	Chitosan/gelatin hydrogels	BMSCs, rabbit	Improves physical–chemical properties, exhibits biocompatibility, and promotes new bone formation	[223]
		Col-DEX-BCP	hMSCs, mouse	Exhibits good porosity, strength, biocompatibility, and osteoinductivity; promotes bone tissue repair	[224]
	MSNs	-	Osteoblasts	Exhibits non-toxicity, high biocompatibility, and adjustable porosity	[226]
		-	Osteoblasts, mouse	Suppresses osteoclast resorption, enhances bone formation and mineralization	[227,228]
		Ti-HA-MSNs	MG-63 cells	Promotes biocompatibility and osteoblast differentiation	[229]
**Magnetics**	Fe_3_O_4_ and γ-Fe_2_O_3_	PLA-HA-γ-Fe_2_O_3_	Osteoblast cells	Improves proliferation, differentiation, and ECM secretion	[234]
		PLA-HA-γ-Fe_2_O_3_	Rabbit	Accelerates bone regeneration in the defect	[235]
		PCL-MNPs	Osteoblast cells, rat	Improves chemical–physical, mechanical, and osteogenic properties; promotes bone regeneration	[236]
		CS/Col/Fe_3_O_4_/nHAP	MC3T3-E1 rat skull osteoblasts/ Rat	Improves cell adhesion and proliferation, as well as osteogenic differentiation in vitro and mineralization and bone regeneration in vivo	[237]
		IONP-CPC	hDPSCs	Improves cell attachment, osteogenic differentiation, and bone mineralization	[238]
**CARBON-BASED**					
**Fullerene**		C60NPEG5000	MG-63	Promotes osteoblast adhesion, proliferation, and differentiation	[244,245,246]
		Nanocrystalline diamond (NCD)	MG-63	Serves as a support for the adhesion, growth, and differentiation of osteogenic cells	[252]
**Nanodiamonds**		ND-ODA/PLLA	SBF	Enhanced mechanical properties and increased mineralization capability	[253]
**Carbon dots**		NCDs-HA	MC3T3-E1, Zebrafish	Promotes osteogenic differentiation in vitro and bone regeneration in vivo	[256]
		PCL/PVA-TCP3-CDs	hBFPSCs	Improves mechanical strength, cell adhesion, and proliferation	[257]
		CD@HAp	MG-63	Improves mechanical strength, cell adhesion, and proliferation	[258]
**Carbon nanotubes**		MWCNTs	MSCs	Allows osteogenic differentiation in vitro, and ectopic bone formation in vivo	[259]
		f-MWCNTs HTAB	HOB	Improves mechanical strength, biocompatibility, and ALP activity	[266]
		CNT/HA	L-929 cells, rabbits	Accelerates cell proliferation and improves mechanical properties	[267]
		MWCNT-coated Col sponge	Saos-2	Improves cell adhesion	[268]
		PLA/MWCNTs	Osteoblast cells	Directs osteoblast outgrowth	[269]
		PLLA/py-end-PLLA/MWCNTs	HBMC	Supports cell adhesion and proliferation and promotes osteogenic differentiation	[270]
		PLGA/c-MWCNT	MSCs	Promotes cell growth and osteoblast differentiation	[271]
		CNT/PLGA	MC3T3-E1	Enhanced surface roughness, increased cell attachment and proliferation	[272]
		MWNTs/PCL	BMSCs	Enhances cell proliferation and differentiation	[274]
		PCL-HA-imCNT	SBF, MC3T3-E1, rat	Improves the compressive strength and elastic modulus; induces substantial mineralization and cell proliferation. Induces vascularization and bone regeneration in vivo	[275]
**Graphene**		Graphene film (GF)	MG-63	Promotes cell adhesion, activity, and the formation of bone-like apatite	[276]
		-	hMSCs	Provides biocompatibility and accelerates bone differentiation	[278]
		HB-3DG-HA	MSCs	Supports cell viability and proliferation, upregulates osteogenic differentiation	[279]
		AG/NG–HA	MSCs	Improves mechanical properties and promotes cell proliferation, viability, and osteogenic differentiation	[280]
		G- or GO-PMMA	MC3-T3	Influences thermal properties and biocompatibility	[281]
		CS/PVP/GO	rbmMSCs, rats	Promotes cell attachment and viability in vitro; in vivo promotes more efficient wound closure	[282]
		nHAp-GO/GA)	MG-63	Enhances compressive strength, reduces the biodegradation rate, and improves mineral deposition	[283]

## Data Availability

No new data were created or analyzed in this study. Data sharing is not applicable to this article.
